# Expression of intracellular toll-like receptors in leukoplakia and oral squamous cell carcinoma

**DOI:** 10.2340/aos.v85.45423

**Published:** 2026-02-11

**Authors:** Divya Ganesh, Jonas Sundberg, Amal Dafar, Göran Kjeller, Dipak Sapkota, Jenny Öhman, Daniel Giglio, Bengt Hasséus

**Affiliations:** a Department of Oral Medicine and Pathology, Institute of Odontology, The Sahlgrenska Academy, University of Gothenburg, Gothenburg, Sweden; b Clinic of Oral and Maxillofacial Surgery, Public Dental Service, Region Västra Götaland, Gothenburg, Sweden; c Department of Oral and Maxillofacial Surgery, King Fahad General Hospital, Jeddah, Kingdom of Saudi Arabia; d Department of Oral and Maxillofacial Surgery, Institute of Odontology, The Sahlgrenska Academy, University of Gothenburg, Gothenburg, Sweden; e Department of Oral Biology, University of Oslo, Oslo, Norway; f Department of Clinical Pathology, Sahlgrenska University Hospital, Gothenburg, Sweden; g Department of Oncology, Institute of Clinical Sciences, The Sahlgrenska Academy, University of Gothenburg, Gothenburg, Sweden; h Department of Medicine and Oncology, Southern Älvsborg Hospital, Borås, Sweden

**Keywords:** oral potentially malignant disorder, oral cancer, toll-like receptors, immune cells, oral leukoplakia

## Abstract

**Objective:**

Toll-like receptors (TLRs) are a group of pathogen recognition receptors expressed not only on immune cells but also cancer cells, where TLR activation may lead to tumour progression or suppression. At present, little is known about the role of TLRs and their connection with immune responses in precancerous lesions, such as oral leukoplakia (OL). In the present study, we have explored the immune activation and the expression of the intracellular TLRs – TLR3, TLR7, TLR8, and TLR9 in OL without and with dysplasia, and in oral squamous cell carcinoma (OSCC).

**Patients and methods:**

Immunohistochemistry was performed on 19 OL patients without dysplasia (OL-no) and 13 patients with dysplasia (OL-dys) and 10 OSCC patients. On digitalised images, TLR3-, TLR7-, TLR8- and TLR9-expressing cells were semi-quantitatively assessed, while the number of CD3- and CD8-expressing cells/mm^2^ was registered.

**Results:**

Nuclear TLR7 appeared in 31% of OL-dys but was absent in OL-no (*p* = 0.03). Cytoplasmic TLR8 was higher in OL-no than OL-dys (32% vs. 8%, *p* = 0.02). Similarly, cytoplasmic TLR9 was also higher OL-no than OL-dys (42% vs. 23%, *p* = 0.01).

**Conclusions:**

TLR3, TLR7, TLR8, and TLR9 are all expressed in OL-no, OL-dys, and OSCC. Also, the study provides evidence for possible nucleocytoplasmic shuttling of TLRs.

## Introduction

In the oral cavity, oral squamous cell carcinomas (OSCC) account for over 90% of all primary oral cancers [1]. OSCC arises from epithelial keratinocytes and can appear as *de novo* tumours, though they frequently develop from precursor lesions in the oral epithelium [2]. One such precursor disorder is oral leukoplakia (OL). OL is classified as an oral potentially malignant disorder (OPMD), with a prevalence of 1% in the population and a malignant transformation rate of 11% [3]*.* The aetiology of OL remains incompletely understood, but mutations and genetic aberrations are of importance, as also exogenous factors such as exposure to tobacco, alcohol, and virus infections [4, 5]. The importance of the immune system in the development of tumours has been well recognised during the last decades [6]. In OSCC, studies on immunological mechanisms have revealed how different immune responses may direct evolvement and outcome of a tumour in the oral cavity [7–11]. Most studies of OSCC focus on the acquired branch of the immune system, but there are also reports on how the innate branch responds to malignant cells [12]. In OPMD such as OL, the situation is much the same, with studies exploring the role of acquired immunity, reporting an increased influx of cytotoxic T cells in OL with dysplasia [11, 13, 14]. However, there is limited knowledge about the activation of the innate branch of the immune system, where toll-like receptors (TLRs) play a central role.

TLRs are a type of pattern recognition receptors that detect pathogen-associated molecular patterns (PAMPs) and danger-associated molecular patterns (DAMPs), triggering an immune response [15, 16]. In humans, 10 TLRs (TLR1-10) have been identified. Among them, TLR3, TLR7, TLR8, and TLR9 are intracellular receptors and are situated on endosomal membranes [17, 18]. TLRs are expressed on immune cells as well as various non-immune cells, including epithelial cells and tumour cells, where they modulate inflammation [19]. As a key component of the innate immune system, TLRs primarily recognise viral and bacterial nucleic acids [20–23], but they also influence tumour cells, promoting either tumour progression or suppression [24, 25]. In OSCC, overexpression of TLR3 has been observed and is associated with high-risk tumours [26, 27]. Conversely, Rusanen et al., reported a lower expression of TLR3, TLR7, and TLR8 in OSCC [12].

Emerging cancer therapies are using TLR agonists in enhancing antitumour response. Intracellular TLRs are, so far, the main target in these treatment strategies, highlighting their importance in influencing cell cycle controlling mechanisms [28]. Studies of TLRs in OL are limited. A study conducted by Kotrashetti indicated that TLR9 expression was low in oral epithelial dysplasia (OED) and moderate in OSCC [29]. Ali et al. reported that TLRs could be detected in OL [30]. But otherwise, no studies have explored this area.

Given that OL represents an intermediary stage between normal epithelium and invasive carcinoma, assessing the expression of TLR3, TLR7, TLR8, and TLR9 in OL and OSCC could provide valuable insights into their roles in disease progression. Since the acquired branch of the immune system is in close interaction with the innate branch, association between TLRs and effector cells such as T cells may also provide valuable insights in immunosurveillance in OL.

Thus, this study aimed to analyse the expression patterns of the intracellular TLRs – TLR3, TLR7, TLR8, and TLR9 – as well as T cell influx in epithelium of OL with or without dysplasia and OSCC.

## Patients and methods

### Patients

A cohort of 32 patients with a clinical diagnosis of OL and a histopathological diagnosis of hyperkeratosis with dysplasia (OL-dys; *N* = 13) and benign hyperkeratosis (OL-no; *N* = 19) were retrospectively retrieved from the archives at the Department of Pathology, Sahlgrenska University Hospital and the Department of Oral Medicine and Pathology, Institute of Odontology, Sahlgrenska Academy. In parallel, 10 patients with a histopathological diagnosis of OSCC were collected from the same archives. The biopsies were formalin-fixed and paraffin-embedded specimens were obtained between 1989 and 2005. Patient characteristics are described in Table 1.

**Table 1:** Patients’ characteristics.

Demographics	OL-no N (%)	OL-dys N (%)	OSCC N (%)
**Patients**	19	13	10
**Age (years)**			
Median; range	64; 44–75	64; 45–91	65; 34–71
Mean	63	66	60
**Gender**			
Male	14 (74)	10 (77)	6 (60)
Female	5 (26)	3 (23)	4 (40)
**Histopathological grading**			
Benign hyperkeratosis	19 (100)		
Mild dysplasia		4 (31)	
Moderate dysplasia		6 (46)	
Severe dysplasia		3 (23)	
Well differentiated OSCC			1 (10)
Moderately differentiated OSCC			5 (50)
Poorly differentiated OSCC			4 (40)
**Site of lesion**			
Floor of the mouth	4 (21)	1 (8)	1 (10)
Buccal mucosa	5 (26)	5 (38)	5 (50)
Tongue	3 (16)	5 (38)	1 (10)
Gingiva	4 (21)	0	3 (30)
Others	3 (16)	2 (16)	0

OL-no: leukoplakia without dysplasia; OL-dys: leukoplakia with dysplasia; OSCC: oral squamous cell carcinoma.

### Immunohistochemistry

Immune staining was performed on formalin-fixed paraffin-embedded tissue specimens, to identify TLR3, TLR7, TLR8, TLR9, CD3, and CD8 molecules expressing cells. Rabbit polyclonal anbodies raised against TLR3 (ab62566; Abcam, Cambridge, UK), TLR7 and TLR8 (PA5-95046, PA5102413; Thermo Fisher, Gothenburg, Sweden), Rabbit monoclonal antibodies raised against CD3 and CD8(MA5-14524, MA5-14548; Thermo Fisher Scientific, Gothenburg, Sweden), and mouse monoclonal antibody raised against TLR9 (ab134368; Abcam, Cambridge, UK) were used. The tissue blocks were cut into 4-μm-thick sections, mounted on electrostatically pre-charged slides, deparaffinised using two changes of xylene, and then rehydrated in 99.5 and 95.0% ethanol for 5 minutes each, followed by washing in distilled water for 10 minutes. Antigen retrieval was carried out by boiling the sections in citrate buffer (pH6) for 35 minutes. After cooling to room temperature, the sections were rinsed in two changes of Phosphate Buffered Saline (PBS), for 5 minutes each time. To block endogenous peroxidase activity, sections were incubated in hydrogen peroxide (0.3%) for 30 minutes. The sections were then rinsed four times in PBS for 5 minutes each time, and non-specific background was blocked by applying PBS that contained 5% goat serum for 20 minutes at room temperature. Sections were then incubated with the primary antibody targeting TLR3 (1:400), TLR7 (1:100), TLR8 (1:50), TLR9 (1:100), CD3 (1:100), CD8 (1:100) at 4°C overnight. Omission of the primary antibodies served as negative controls. As positive controls, sections from healthy controls were used [30]. On the second day, the sections were washed twice with PBS for 5 minutes each, and then incubated for 1 hour at room temperature with secondary goat anti-mouse horseradish peroxidase (HRP) antibody (1:500; ab6789; Abcam, London, UK), secondary goat anti-rabbit HRP antibody (1:500; ab6721; Abcam, London, UK), and secondary goat anti-rabbit antibody biotin (1:1000; B2770; Thermo Fisher Scientific, Gothenburg, Sweden). This was followed by two washes with PBS for 5 minutes each, the sections were then incubated with Avidin-Biotin Complex (ABC) staining solution for 30 minutes. The sections were then rinsed two times in PBS for 5 minutes each time, and were then incubated with 3,3’-Diaminobenzidine (DAB) substrate for 10 minutes. The sections were thereafter rinsed two times in PBS for 5 minutes, added with hematoxylin for 2 minutes, and rinsed with running warm tap water for 3 minutes. This was followed by dehydration in 95.0 and 99.5% ethanol for 5 minutes each, and two changes of xylene for 5 minutes each. Thereafter, the sections were mounted with cover glasses.

To assess Candida infection, Periodic Acid-Schiff (PAS) staining was performed at the Department of Pathology, Sahlgrenska University Hospital.

### Semi-quantitative and quantitative analysis

Digitalised images of stained sections were captured with a light microscope at magnification x200 (Leitz Wetzlar, Leica Microsystems, Wetzlar, Germany) and digital camera (UC30; Olympus, Microsystem, Norcross, GA, USA). In OL-no sections, two to three high-power fields (HPFs) were selected, including one central and two peripheral areas in the epithelium. In OL-dys sections, two to three HPFs were selected specifically from dysplastic regions. In OSCC sections, three randomly selected tumour islands were chosen. QuPath (version 0.2.0-m4) software was used for the quantification of positive cells [31].

**Figure 1:**
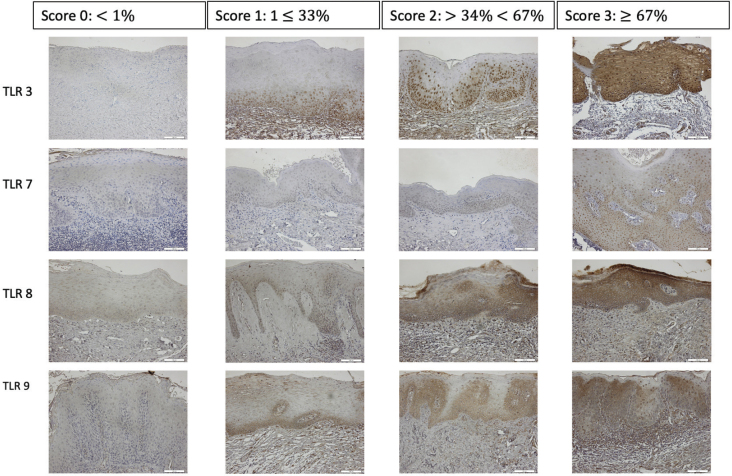
TLRs scoring scale. Semi-quantitative assessment of TLR3, TLR7, TLR8 and TLR9 expressions in leukoplakia based on the percentage of TLR expression in oral epithelium. Magnification: ×200. TLR: Toll-like receptors.

**Figure 2:**
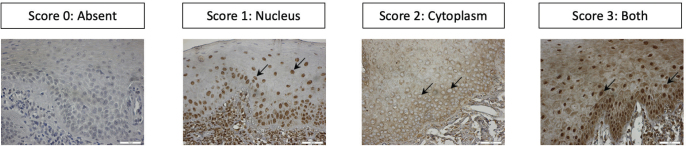
TLRs categorical assessment of TLR3, TLR7, TLR8 and TLR9 expressions in leukoplakia based on nuclear, cytoplasmic expression (arrows) in oral epithelium. Magnification: ×400. TLR: Toll-like receptors.

To semi-quantitively assess TLR3-, TLR7-, TLR8- and TLR9-expression in the epithelium of OL, a scoring scale was adopted where absence of positive cells was graded 0, 1 to ≤33% positive cells was graded 1, >34 to <67% was graded 2 and ≥67% was graded 3, and the mean score from images for each patient was registered (Figure 1). In OSCC, semi-quantitative assessment of three tumour islands was done with the same scoring scale, and the mean score from three images for each patient was registered. The nuclear and/or cytoplasmic expression scores staining of TLR3, TLR7, TLR8, and TLR9 was semi-quantitatively assessed and recorded as Neg: negative, Nuc: positive nuclei, Cyt: positive cytoplasm, and Nuc & Cyt: positive nuclei and cytoplasm. In OL-no and OL-dys, the highest score for each patient was registered (Figure 2). In OSCC, semi-quantitative assessment of three tumour islands was done with the same scoring scale, and the highest score from three images for each patient was registered. To quantitatively assess the number of CD3- and CD8-expressing cells, positively stained nucleated cells in the epithelium from one central HPF field were counted manually on digitalised images using the Qupath computer software and numbers of positive cells/mm^2^ were registered. This assessment was carried out by two independent observers (AD, JS), calibration was done before the assessment and in the case of discrepancy between observers, a third observer (DG) assessed the tissue sections.

### Statistical analysis

The Kruskal-Wallis test (or H test) was used to compare antigen expressions between OL-no, OL-dys, and OSCC. The Fisher’s exact test was used to analyse nuclear and/or cytoplasmic expression in OL-no and OL-dys. The statistical software GraphPad Prism version 9.2.0 software (GraphPad Inc., San Diego, CA, USA) was used for analysis. *P*-values of <0.05 were considered as statistically significant.

### Ethical approval

The study was approved by the Swedish Ethical Review Authority (Dnr: 2019-04579, 2020-04405). In their approval, the Swedish Ethical Review Authority waived informed consent from the patients since this is a retrospective study on historical patients over a long time period.

## Results

### TLR3-, TLR7-, TLR8- and TLR9-expression in leukoplakia with and without dysplasia and squamous cell carcinoma

**Figure 3:**
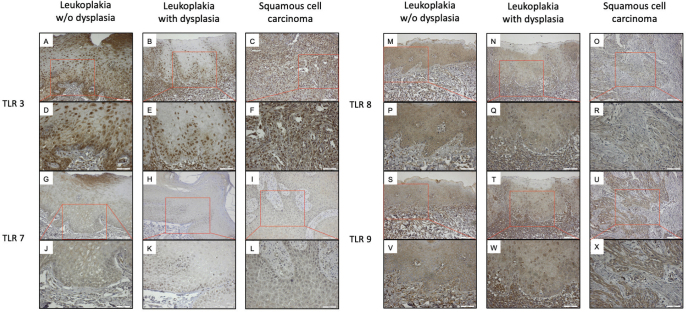
TLR3-positive cells (brown) in OL-no (A, D), OL-dys (B, E), and OSCC (C, F); TLR7-positive cells in OL-no (G, J), OL-dys (H, K), and OSCC (I, L); TLR8-positive cells in OL-no (M, P), OL-dys (N, Q), and OSCC (O, R); TLR9-positive cells in OL-no (S, V), OL-dys (T, W), and OSCC (U, X) shown at × 200 and × 400 magnification. TLR: Toll-like receptors; OL-no: leukoplakia without dysplasia; OL-dys: leukoplakia with dysplasia; OSCC: oral squamous cell carcinoma.

In epithelial keratinocytes of OL-no and OL-dys as well as in OSCC cells, TLR3 (Figure 3A–C), LR7 (Figure 3G-I), TLR8 (Figure 3M-O), and TLR9 (Figure 3S-U) were detected. In OSCC, both nuclear & cytoplasmic positivity for TLR3 (Figure 3F) and TLR7 (Figure 3L) were detected. TLR8-positive cells in OL-no, OL-dys, and OSCC cases showed a varying degree of nuclear & cytoplasmic positivity (Figure 3P–R). TLR9-positive cells in OL-no and OSCC cases with solely cytoplasmic or cytoplasmic & nuclear positivity were detected (Figure 3V–X).

**Figure 4:**
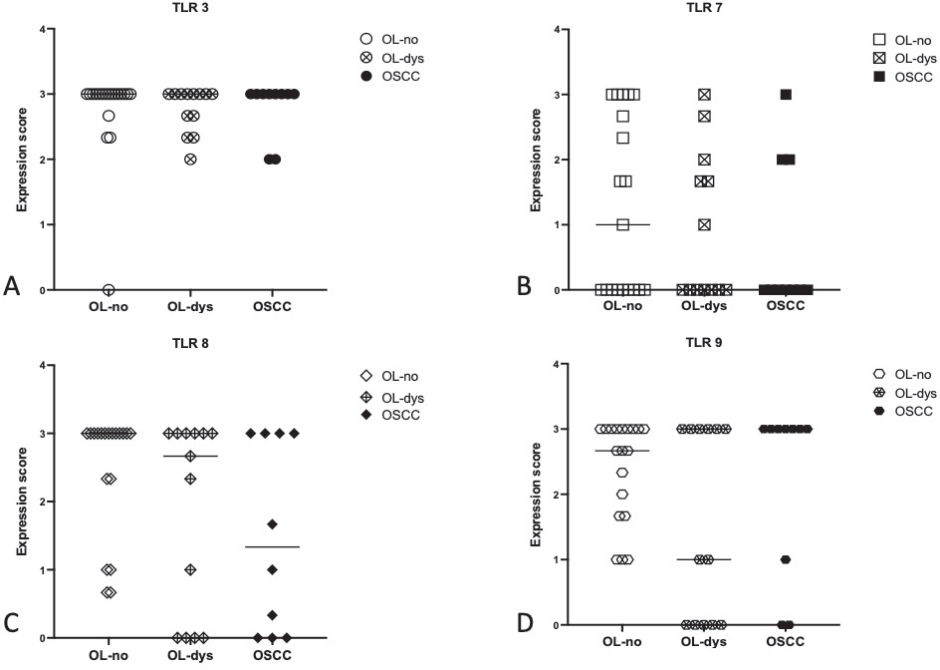
Expression scores in OL-no, OL-dys and OSCC for TLR3 (A), TLR7 (B), TLR8 (C), TLR9 (D). TLR: Toll-like receptors; OL-no: leukoplakia without dysplasia; OL-dys: leukoplakia with dysplasia; OSCC: oral squamous cell carcinoma.

A comparison of TLR3-, TLR7-, TLR8-, and TLR9-expressing cells did not show any significant differences between OL-no and OL-dys patients (*p* = 0.99, *p* = 0.99, *p* = 0.39, and *p* = 0.61, respectively; Figure 4), no significant difference in TLR3, TLR7, TLR8, and TLR9 was noted in OL-no and OSCC (*p* = 0.99, *p* = 0.70, *p* = 0.18, and *p* = 0.99, respectively; Figure 4), and similarly no significant difference was noted between OL-dys patients and OSCC (*p* = 0.99, *p* = 0.99, *p* = 0.99, and *p* = 0.57, respectively; Figure 4)

**Figure 5:**
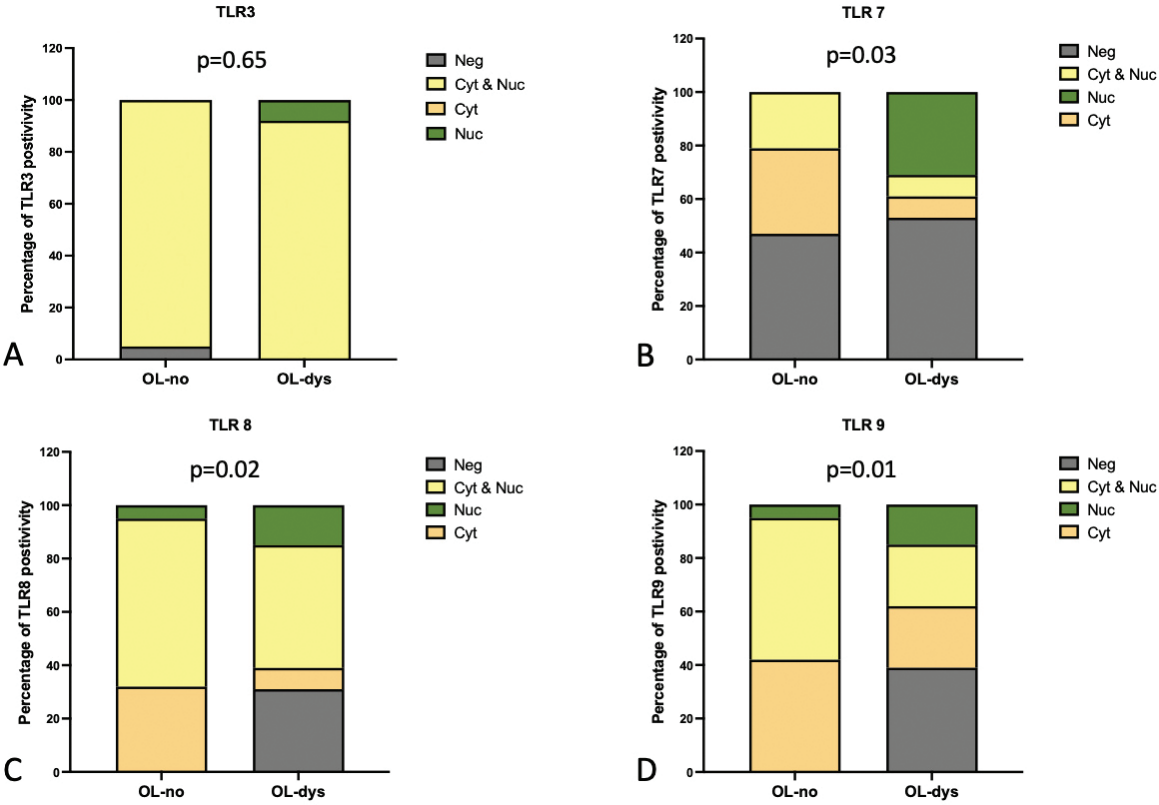
Nuclear and/or cytoplasmic expression scores in OL-no and OL-dys for TLR3 (A), TLR7 (B), TLR8 (C) and TLR9 (D). TLR: Toll-like receptors; OL-no: leukoplakia without dysplasia; OL-dys: leukoplakia with dysplasia; OSCC: oral squamous cell carcinoma.

Nuclear and/or cytoplasmic expression of TLR3, did not reveal any significant differences between OL-no and OL-dys (*p* = 0.65; Figure 5A). Nuclear TLR7 expression was absent in OL-no but present in 31% (*n* = 4) of OL-dys cases (*p* = 0.03; Figure 5B). Cytoplasmic TLR8 expression was observed in 32% (*n* = 6) of OL-no and 8% (*n* = 1) of OL-dys cases (*p* = 0.02; Figure 5C), while cytoplasmic TLR9 expression was observed in 42% (*n* = 8) of OL-no and 23% (*n* = 3) of OL-dys cases (*p* = 0.01; Figure 5D).

The results from PAS staining revealed one OSCC patient positive for candida infection.

### CD3- and CD8-expressing cells in leukoplakia with and without dysplasia

**Figure 6:**
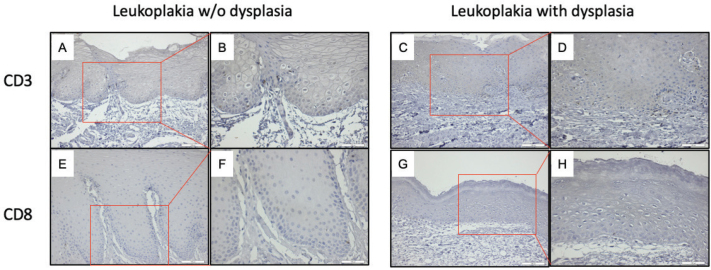
CD3 positive cells (brown) in OL-no (A, B), OL-dys (C, D); CD8 positive cells (brown) in OL-no (E, F), OL-dys (G, H) shown at ×200 and ×400 magnification. OL-no: leukoplakia without dysplasia; OL-dys: leukoplakia with dysplasia; OSCC: oral squamous cell carcinoma.

**Figure 7:**
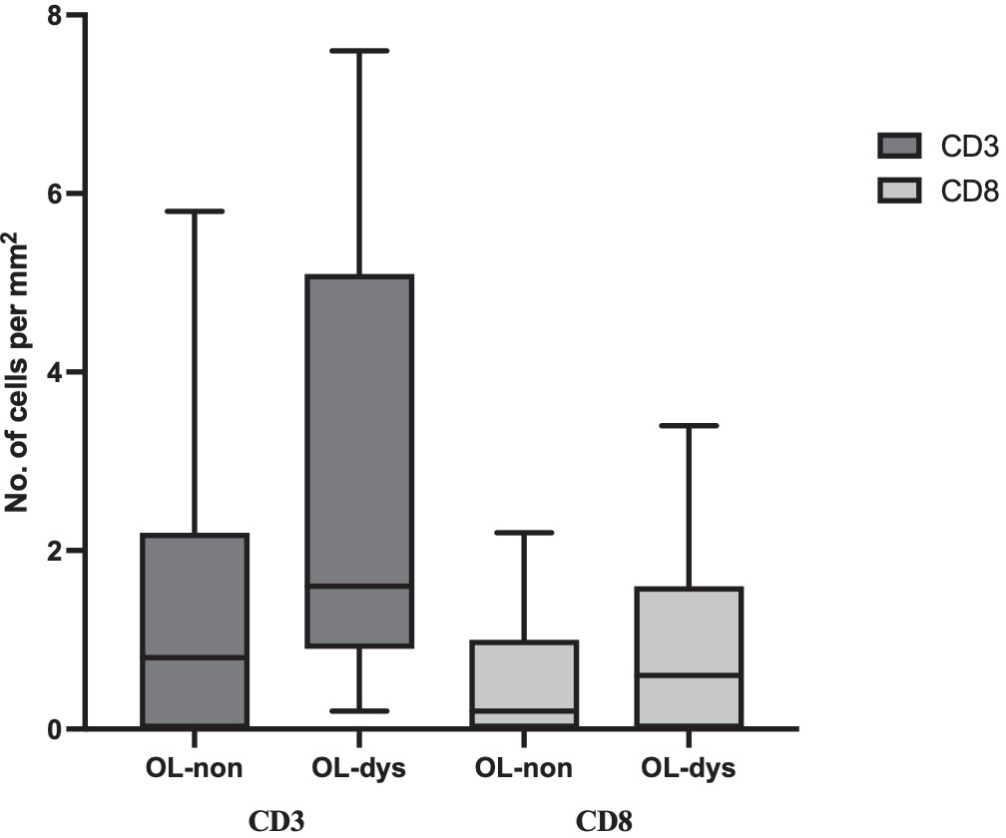
Number of CD3 and CD8 positive cells/mm2 in OL-no and OL-dys. OL-no: leukoplakia without dysplasia; OL-dys: leukoplakia with dysplasia.

CD3 and CD8-expressing lymphocytes localised predominantly in the stratum basale and spinosum layer of the epithelium of OL-no and OL-dys (Figure 6A–H). CD3 expressing T cells did not differ significantly between the OL-dys and OL-no (*p* = 0.11; Figure 7). The number of CD8 expressing lymphocytes did not differ between the OL-dys and OL-no (*p* = 0.49; Figure 7).

## Discussion

The study shows that TLR3, TLR7, TLR8, and TLR9 are expressed in the epithelium of OL patients with or without dysplasia and also in OSCC. In TLR7, increased nuclear expression could be seen in OL-dys compared with OL-no. TLR8 and TLR9 cytoplasmic expressions were decreased in OL-dys compared to OL-no.

Few studies have explored the nuclear and cytoplasmic localisation of TLRs in malignant transformation. Kauppila et al. showed that TLR5 expression shifted to a more diffused expression during the progression from normal to dysplasia and to oropharyngeal squamous cell carcinoma (OPSCC) [32]. In another study, the cytoplasmic expression of TLR5 in oral cancer was higher compared to oral epithelium from healthy controls [33]. Previous studies showed that TLR3 and TLR9 were detected on both the plasma membrane and in the cytoplasm of OPSCC cases, while TLR7 was found on nuclear membranes and/or in the nuclei of OPSCC cases [34]. In our study, TLR3, TLR7 in OL-dys were found mostly in the nuclei alone or in both the nuclei and the cytoplasm. On the other hand, TLR9, TLR8 in OL-no were either mostly present only in the cytoplasm or both the nuclei and cytoplasm.

The definite cellular localisation of a protein is important in regulation of proliferation and apoptosis [35]. Proteins may be shuttled between the cytoplasm and the nucleus, which is a phenomenon known as nucleocytoplasmic shuttling [35]. Different mechanisms are involved in this transportation; one such important mechanism is transport signals for nuclear import and export [35]. Speculatively, based on our results, TLR7 may be shuttled from the cytoplasm into the nucleus thereby activating downstream signalling pathways to activate proliferation of dysplastic cells. In contrast, in OL-no patients TLR7 expressed in an inactive state in either the cytoplasm or both the nuclei and the cytoplasm without any shuttling. On the other hand, shuttling was less evident for TLR9 in OL-dys patients, since it was either absent or mostly expressed in either only the cytoplasm or both the nuclei and the cytoplasm, whereas in OL-no it was expressed only in the cytoplasm or both, indicating absence of shuttling to the nucleus. To explore the nucleocytoplasmic shuttling, we need to understand the signalling pathway involved in the TLRs. As a reference to OL, we show that the expression of TLR3, TLR7, TLR8, and TLR9 in OSCC is in line with our previous results and the results of other research groups [12, 36].

As part of the innate immune system, TLRs influence the acquired immune response and their effector cell where T cells are key players [37]. In this study, there was a trend of higher numbers of CD3-expressing T cells in OL-dys compared to OL-no patients, whereas CD8 expressing cells did not show any significant difference between the groups. Öhman et al. reported increased number of CD3 positive and CD8 positive lymphocytes in patients with OL-dys in comparison to OL-no [11]. Although this study did not confirm a significant difference, a similar trend with influx of CD3 positive T cells was registered, indicating an activation of the acquired branch of the immune system.

Candida infections were rare in our cohort of patients and only one OSCC patient was positive in PAS-staining. Since candida infection triggers a TLR response [30], absence of candida in the analysed specimens implicate that fungal infection does not influence our results.

TLRs have emerged as target molecules in cancer therapy in order to modulate the tumour microenvironment [28]. Several clinical studies are underway using agonists against all the intracellular TLRs in different cancer types including one study in oral cancer utilising the TLR7 agonist imiquimod [38, 39]. Thus, increased knowledge about TLR expression in OPMD such as OL are warranted, and may open possibilities to treat, at least high-risk OL patients, with TLR-modulating molecules.

A limitation of the present study was the retrospective design which curtails retrieval of detailed clinical and anamnestic data. In addition, the number of patients in the study cohort was limited, which affects the power of performed analyses. In the study we focused on presence of TLR3, TLR7, TLR8, and TLR9 in keratinocytes of the oral epithelium and did not assess immune cells present in the subepithelial compartment. Future exploration of this tissue compartment will probably disclose more knowledge about the OL microenvironment, but larger cohorts are needed to address this. It would also be desirable to assess the TLR expression in relation to the degree of dysplasia in OL and degree of differentiation in OSCC, but these sub-analyses are not powered by the number of patients.

In conclusion, in this retrospective study we show that TLR3, TLR7, TLR8, and TLR9 are expressed in OL. We also present evidence for possible nucleocytoplasmic shuttling of TLR7, TLR8 and TLR9 in OL with dysplasia.
